# Effect of Threading Dislocations on the Electronic Structure of La-Doped BaSnO_3_ Thin Films

**DOI:** 10.3390/ma15072417

**Published:** 2022-03-25

**Authors:** Jeonghun Kang, Jeong Hyuk Lee, Han-Koo Lee, Kwang-Tak Kim, Jin Hyeok Kim, Min-Jae Maeng, Jong-Am Hong, Yongsup Park, Kee Hoon Kim

**Affiliations:** 1 Center for Novel States of Complex Materials Research, Department of Physics and Astronomy, Seoul National University, Seoul 08826, Korea; kangjh1127@snu.ac.kr (J.K.); xaaer5@snu.ac.kr (J.H.L.); kgtakee@snu.ac.kr (K.-T.K.); 2 Pohang Accelerator Laboratory, Pohang University of Science and Technology, Pohang 37673, Korea; hangulee@postech.ac.kr; 3 Optoelectronic Convergence Research Center, Department of Materials Science and Engineering, Chonnam National University, Gwangju 61185, Korea; jinhyeok@chonnam.ac.kr; 4 Department of Physics, Kyung Hee University, Seoul 02447, Korea; dut0618@khu.ac.kr (M.-J.M.); elavian@khu.ac.kr (J.-A.H.); parky@khu.ac.kr (Y.P.); 5 Department of Information Display, Kyung Hee University, Seoul 02447, Korea; 6 Department of Physics and Astronomy, Institute of Applied Physics, Seoul National University, Seoul 08826, Korea

**Keywords:** perovskite oxide, barium stannate (BaSnO3), photoemission spectroscopy, threading dislocation, bandgap renormalization

## Abstract

In spite of great application potential as transparent *n*-type oxides with high electrical mobility at room temperature, threading dislocations (TDs) often found in the (Ba,La)SnO_3_ (BLSO) films can limit their intrinsic properties so that their role in the physical properties of BLSO films need to be properly understood. The electrical properties and electronic structure of BLSO films grown on SrTiO_3_ (001) (STO) and BaSnO_3_ (001) (BSO) substrates are comparatively studied to investigate the effect of the TDs. In the BLSO/STO films with TD density of ~1.32 × 10^11^ cm^−2^, *n*-type carrier density *n*_e_ and electron mobility are significantly reduced, as compared with the BLSO/BSO films with nearly no TDs. This indicates that TDs play the role of scattering-centers as well as acceptor-centers to reduce *n*-type carriers. Moreover, in the BLSO/STO films, both binding energies of an Sn 3*d* core level and a valence band maximum are reduced, being qualitatively consistent with the Fermi level shift with the reduced *n*-type carriers. However, the reduced binding energies of the Sn 3*d* core level and the valence band maximum are clearly different as 0.39 and 0.19 eV, respectively, suggesting that the band gap renormalization preexisting in proportion to *n*_e_ is further suppressed to restore the band gap in the BLSO/STO films with the TDs.

## 1. Introduction

In recent years, the perovskite stannate BaSnO_3_ (BSO) system with donor-doping (e.g., La^3+^) has received considerable attention due to its high electron mobility (*μ_e_*), high electrical conductivity (*σ*), high optical transparency, and excellent thermal/chemical stability. Moreover, with the control of La^3+^ doping levels, the BSO system can play versatile roles of either transparent semiconductors or transparent conductors. Various transparent electronic devices employing the La-doped BSO (Ba_1*–x*_La*_x_*SnO_3_, BLSO) have been demonstrated: active *n*-channel materials in the *p*-*n* junctions [1] and the field-effect transistors (FETs) [2], sensitive UV photoconductors [3], and electron transport layers in perovskite solar cells [4].

Among the various forms of BLSO, single crystals have so far exhibited the highest *μ_e_*~320 cm^2^/V∙s and the highest *σ*~2 × 10^4^ S/cm at room temperature. To find the origin of these superior electrical characteristics, the band structure of the BLSO film has been intensively investigated both theoretically and experimentally. The indirect nature of the bandgap ~3.1 eV and conduction band filling of the BLSOs were observed by various experimental procedures such as X-ray photoemission spectroscopy (XPS), optical spectroscopy, and ellipsometry [5,6,7]. Corroborating the experimental results, several recent calculations on the band structures of BLSO with density functional theory (DFT) combined with the hybrid functions predict that the La-doping into BSO results in a degenerate semiconductor with an indirect bandgap of ~3.1 eV [8,9,10,11]. Moreover, typical behavior of the degenerate semiconductor such as the Burstein–Moss shift and band gap renormalization was observed in the BLSO films [6]. Both theoretical and experimental investigations indicate that the superior electrical characteristics mainly stem from two intrinsic physical properties. First, the BSO allows the degenerate-doping of La^3+^ ions into a highly dispersive conduction band, mainly composed of Sn 5*s* orbitals, allowing a full activation of donors and a small effective mass of electron carriers. Secondly, a high dielectric constant (~20) of BSO leads to the suppressed ionic dopant scattering, resulting in a high *μ_e_* [11].

However, the electrical properties of BLSO films are often inferior to those of single crystals. For example, the BLSO films grown on the SrTiO_3_ (STO) (001) substrate, having a lattice mismatch of about ~−5.4%, exhibit a typical *μ_e_*~10–70 cm^2^/V∙s [12]. This is mainly due to the additional carrier scattering coming from the various types of defects or dislocations, i.e., oxygen vacancies, Ba/Sn antisites, Ruddlesden–Popper shear faults, and threading dislocations (TDs) [13,14,15,16,17,18]. To envision further improvement of the *μ_e_* of the BLSO films, it is thus essential to control the various types of defects and understand the role of such defects in the physical properties of the films.

Toward the end of realizing the films with higher *μ_e_*, there have been various experimental efforts such as different growth methods, the use of buffer layers and substrates with reduced lattice mismatch, and post-treatment. All of those efforts have so far resulted in the highest achievable *μ_e_*~70–183 cm^2^/V∙s [13,14,15,16,17,18], which are overall improved but still scattered. As an example relevant to the present study, Mun et al. [19] controlled the growth temperature of Ba_0.96_La_0.04_SnO_3_/STO (001) films and revealed that *μ_e_* is enhanced when TD density (*N*_D_) is reduced. Furthermore, with the control of the post-annealing condition at Ar and H_2_ atmosphere, Yoon et al. [20] similarly observed that the *μ_e_* of Ba_1−*x*_La*_x_*SnO_3_/MgO (001) films, having a lattice mismatch of ~+2.2% between *a*_BSO_ = 4.116 Å and *a*_MgO_ = 4.212 Å, is proportional to 1/*N*_D_. The highest *μ_e_* at room temperatures achieved in those studies were ~70 cm^2^/V∙s (*n_e_* = 4.0 × 10^20^ cm^−3^) [19] and 120 cm^2^/V∙s (*n_e_* = 1.1 × 10^20^ cm^−3^) [20], respectively. Both studies have thus suggested a critical role of TD in limiting *μ_e_*. On that account, controlling TD seems to be crucial for improving the *μ_e_* of the BLSO films.

The actual *μ_e_* should also be affected by other defect sources, which were simultaneously created by either growth temperatures, annealing atmosphere, or the La^3+^ dopant concentration. For example, Yoon et al. [20] observed from the XPS study of the O 1s core level that extra oxygen vacancies were formed in the BLSO films upon being post-annealed under H_2_ gas. Moreover, Cho et al. [21] observed that extra oxygen vacancies were created with increasing La^3+^ dopant concentration from the XPS study of the O 1s core level. Therefore, understanding the pure effects of the TD only on the transport properties is a challenging task. Moreover, how the electronic structures of BLSO films are affected by the TD has not been fully understood either as the specimens with the TD only are still lacking.

In this regard, the use of a BaSnO_3_ (BSO) (001) substrate (with a lattice mismatch < 0.03%) in the growth should be one of the most effective ways to achieve the BLSO film without TD. In our former study [15], a Ba_0.99_La_0.01_SnO_3_ film grown on BSO (001), subsequently followed by in-situ O_2_ annealing, resulted in no TD, *μ_e_*~100 cm^2^/V∙s and *n_e_* = 1.3 × 10^20^ cm^−3^ at room temperature. On the other hand, upon the same growth and in-situ annealing conditions being applied, a Ba_0.99_La_0.01_SnO_3_/STO (001) film showed *μ_e_*~25 cm^2^/V∙s and *n_e_* = 5.8 × 10^19^ cm^−3^ [22]. These two BLSO films grown on BSO (001) and STO (001) with the same La^3+^ dopant concentration and growth/annealing condition are thus expected to be useful for comparing the physical properties of the BLSO films with or without TDs.

In this work, we investigate the role of TD on the electrical properties and electronic band structure of the BLSO films. To have the two kinds of films with or without TDs, the BLSO films with 0.5%, 1%, and 4% La doping are grown under identical growth and annealing conditions on two different substrates, BSO (001) (*a* = 4.116 Å) and STO (001) (*a* = 3.905 Å). Through the use of the BSO (001) substrate, the BSLO films without TDs are successfully obtained. Therefore, these two sets of BLSO films grown on STO (001) and BSO (001) could be an interesting platform to trace the intrinsic effect of TDs on their transport properties and electronic structures.

## 2. Materials and Method

### 2.1. Sample Preparation

Ba_1−*x*_La*_x_*SnO_3_ films with *x* = 0.005, 0.01, and 0.04, all of which having a thickness *t* = ~80 nm, were grown on the BSO (001) and the STO (001) substrate by the pulsed laser deposition (PLD) method, the growth conditions of which have been reported previously [15]. Stoichiometric dense polycrystalline pellets of Ba_1−*x*_La*_x_*SnO_3_ with *x* = 0.005, 0.01, and 0.04 were synthesized to be used as the target material in the PLD process. To minimize the variation of the film quality, the films were grown under the same experimental conditions: a 10 Hz repetition rate by the KrF laser (wavelength 248 nm), laser fluence of 0.7 J/cm^2^, oxygen partial pressure of 100 mTorr, and the substrate temperature of 790 °C.

A commercial STO (001) (MTI Corp., Richmond, VA, USA, *t* = 0.5 mm) and a custom-made BSO (001) single crystal were used as the substrates. The BSO single crystal was grown by the flux method using the mixture of CuO and CuO_2_ as a flux, as described in our previous report [15]. Then, the (001) surface of the as-grown BSO crystal was polished using a commercial polishing machine (Allied #70-1218) with polishing cloths and colloidal silica suspension to achieve a smooth surface. The polished BSO (001) substrate exhibited excellent crystallinity as indicated by the full width at half maximum (FWHM) of 0.022° in the *ω*-rocking curve of the (002) peak. Moreover, the root-mean-square roughness of 0.09 nm was achieved after polishing to prove an atomically smooth surface [15].

### 2.2. Structure and Electrical Properties Analysis

The structural properties of the BLSO films were investigated by the reciprocal space mapping (RSM) using high-power X-ray diffractometers (Empyrean^TM^, PANalytical, Malvern, UK). All the RSM data in this study were measured near the BLSO (103) peak. To visualize the TD, transmission electron microscope (TEM) images (JEM-3000F, JEOL, Tokyo, Japan) were obtained in the two Ba_0.96_La_0.04_SnO_3_ films grown on STO (001) and BSO (001). Electronic properties including resistivity (*ρ*), *n_e_*, and *μ_e_* were investigated by the Hall effect measurement system (HL5500PC, Bio-Rad, Bend, OR, USA) with Van der Pauw contacts at room temperature. To ensure ohmic contact, Ti (thickness *t* = 5 nm) and Au (*t* = 50 nm) electrodes were sequentially deposited by a custom-made thermal evaporator.

### 2.3. Photoemission Spectroscopy Measurement

To investigate the electronic structure of BLSO films, ultraviolet photoemission spectroscopy (UPS) and XPS based on the synchrotron radiation source were performed at the Pohang Accelerator Laboratory (PLS-Ⅱ4D, photoemission beamline). Each sample was electrically connected to the ground by using a carbon tape to prevent the surface charging effects. Inside the chamber with the ultra-high-vacuum condition, each sample was then etched with Ar plasma for ~30 s to remove surface contamination. Before XPS and UPS measurements, to achieve accurate electron binding energy (*E*_B_), the work function of the spectrometer was calibrated using the Au reference. Photon energies used for XPS and UPS were 650 eV and 90 eV, respectively.

## 3. Result and Discussion

In our previous study [15], it was established that Ba_1−*x*_La*_x_*SnO_3_ (*x* = 0.00–0.04) films with *t* = ~90 nm could be epitaxially grown on the BSO (001) substrate without any dislocation and with minimized strain development. On the other hand, those grown on STO films with *t* = ~100 nm have exhibited many TDs with fully relaxed strains [19]. Here, with nearly the same growth conditions, including the same oxygen partial pressure and temperatures, we have grown the Ba_1−*x*_La*_x_*SnO_3_ (*x* = 0.005, 0.01, and 0.04) films on the two kinds of substrates, STO (001) and BSO (001). Moreover, we have also fixed *t* = 80 nm in both sets of films as the transport properties are known to be also varied by thickness [23] via, e.g., strain. In this way, we presume that the transport and the electronic structure can be most affected by the TD, and the influences by other defects or strains can be minimized.

**Figure 1:**
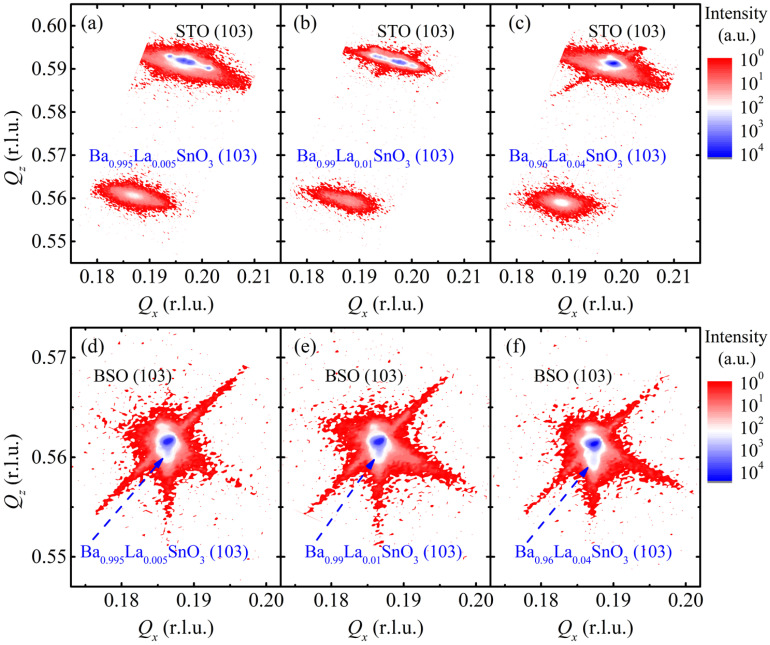
The reciprocal space mapping (RSM) data of Ba_1−*x*_La*_x_*SnO_3_ (*x* = 0.005, 0.01, 0.04) films grown on (**a**–**c**) STO (001) and (**d**–**f**) BSO (001) subtrates, all of which were taken near the (103) peaks.

Figure 1 presents the RSM data measured in the Ba_1−*x*_La*_x_*SnO_3_ (*x* = 0.005, 0.01, and 0.04) films grown on STO (001) and BSO (001). The RSM data in Figure 1a–c show that the BLSO (103) peak is well separated from the STO (103) peak, which directly supports the fully relaxed strains across the BLSO/STO films, regardless of the La doping ratio. On the other hand, as found in Figure 1d–f, the BLSO (103) peaks of the BLSO/BSO films are located at almost the same reciprocal positions with the corresponding peaks of BLSO/STO films. Moreover, they overlapped well with the BSO (103) peak so that they are not even distinguishable from the BSO (103) peak. Note that the BLSO/BSO (103) peaks are slightly elongated along the *Q_z_* direction as the La doping ratio *x* increases. However, such a small strain variation within *t* = 80 nm is not likely to affect the transport properties significantly. All these observations in the RSM data thus support the conclusion that the nearly perfect in-plane lattice match resulted in the BLSO/BSO films with minimal effect of strain variation, and their lattice constants are similar to those of BLSO/STO films.

**Figure 2:**
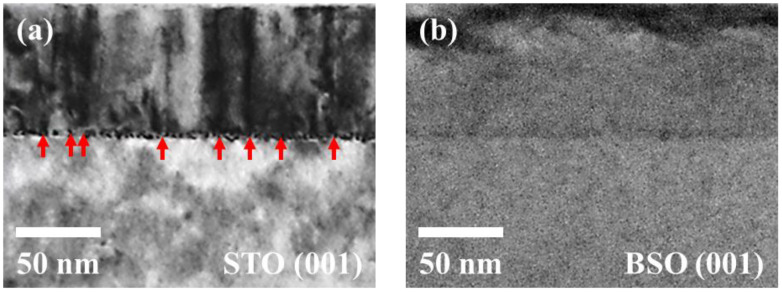
Transmission electron microscope (TEM) images of Ba_0.96_La_0.04_SnO_3_ film deposited on (**a**) STO (001) and (**b**) BSO (001) substrates. Red arrows in (**a**) indicate the TDs.

To visualize the possible TD and estimate its areal density *N*_D_, a cross-sectional TEM study was performed, particularly in the Ba_0.96_La_0.04_SnO_3_ films grown on STO (001) and BSO (001) substrates (Figure 2a,b). The TDs can be easily identified as dark lines in the TEM image of the BLSO/STO (Figure 2a, red arrows). To extract *N*_D_, we have counted the number of TDs with more than 50 % thickness in a wide range TEM image (Figure S1). The number is then divided by a lateral dimension of the image to calculate the line density, of which square was used to estimate the areal density *N*_D_ as ~1.32 × 10^11^ cm^−2^. Note that *N*_D_ is, in principle, expected to be varied with the lattice constant variation of the film. However, as the lattice constants of the three BLSO films (*x* = 0.005, 0.01, and 0.04) are nearly the same within 0.03%, which is also obvious in the RSM data in Figure 1a–c, the *N*_D_ values of the other Ba_1−*x*_La*_x_*SnO_3_/STO films (*x* = 0.005 and 0.01) are likely similar to that of the Ba_0.96_La_0.04_SnO_3_/STO film.

In sharp contrast to the Ba_0.96_La_0.04_SnO_3_/STO films, no TDs can be found in the Ba_0.96_La_0.04_SnO_3_/BSO film, as is evident in Figure 2b. This is understood to be due to the nearly perfect in-plane lattice match between the film and the substrate. Therefore, a comparison of physical properties in the two sets of BLSO films grown on BSO and STO substrates with *t* = 80 nm can be suitable to understand the effect of TD with a relatively low strain effect involved. We emphasize again that in all the RSM data of Figure 1, the center position of each BLSO/BSO (103) peak is very close to the corresponding center position of the BLSO/STO (103) peak. This indicates that the physical properties within the BLSO grains at least could be similar to each other, regardless of the substrate used.

**Figure 3:**
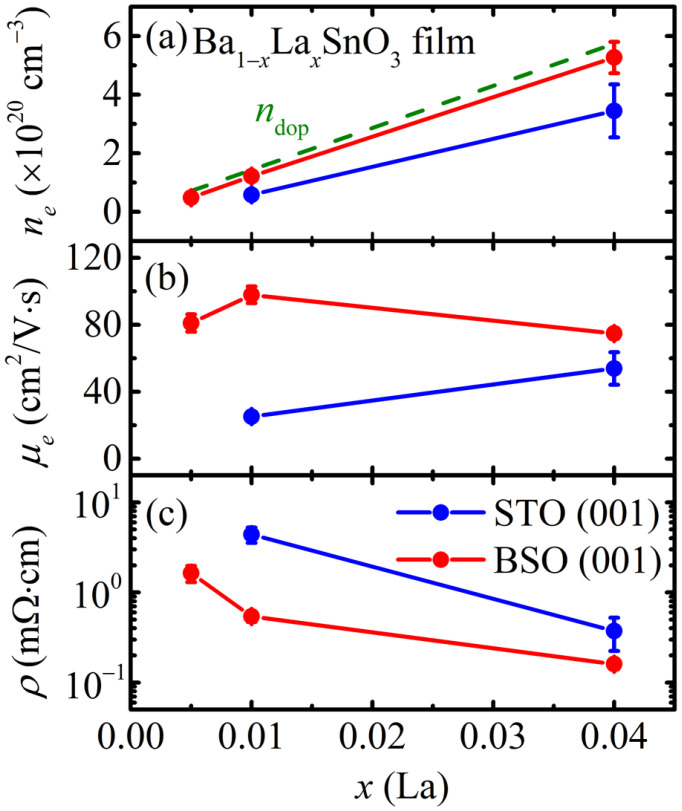
(**a**) Electron concentration (*n_e_*), (**b**) electron mobility (*μ_e_*), and (**c**) resistivity (*ρ*) of the Ba_1−*x*_La*_x_*SnO_3_ films grown on BSO (001) (red solid symbols) and STO (001) (blue solid symbols) substrates. A green dotted line in (**a**) indicates a nominal dopant concentration (*n*_dop_) expected from the fully activated La^3+^ dopant.

Figure 3 compares the electrical properties of the BLSO films with various La doping ratios (*x* = 0.005, 0.01, and 0.04), grown on both STO (001) and BSO (001) substrates. Note that the electrical properties of the Ba_0.995_La_0.005_SnO_3_/STO film could not be measured due to its high resistivity. In Figure 3, two major systematic behaviors can be identified. For all *x*, BLSO/STO films exhibit lower *n_e_* and *μ_e_* as compared with BLSO/BSO films. These experimental findings indicate that the electrical properties of BLSO films are affected by the TDs in two ways. First, TDs act as acceptor-centers and thus reduce *n_e_*. Secondly, TDs provide extra scattering centers and thus reduce *μ_e_*. A similar reduction of *μ_e_* limited by the dislocation was also reported in the other BLSO films (*μ_e_*~*N*_D_^−1^) [20] and binary semiconductors such as GaN and GaAs [24].

The reduced *n_e_* and *μ_e_* in the BLSO/STO films can be explained by the role of dangling bonds existing at TDs. The periodic lattice structure of the BLSO film is expected to be discontinued at the TDs, which can naturally lead to the dangling bonds in the atoms located at the dislocation sites. These dangling bonds can then attract free electrons near the TDs and trap them into their bonding sites, thus resulting in much reduced *n_e_* as compared with that of the BLSO/BSO films (Figure 3a). From the TEM image in Figure 2a, one can assume that each TD can be approximately modeled as a square rod with a height of 80 nm and width of 10 nm, which results in ~1.89 × 10^4^ dangling bonds per one TD if each unit cell contains one dangling bond. With the observed *N*_D_ = 1.32 × 10^11^ cm^−2^, a maximum reduced *n_e_* of 3.12 × 10^20^ cm^−3^ is then expected for the Ba_0.96_La_0.04_SnO_3_/STO film (see Supplementary Information for details). Note that the experimental value of the reduced *n_e_* in Figure 3a is 2.29 × 10^20^ cm^−3^ for the La 4% specimen, which is roughly consistent with the estimated value. Furthermore, upon the free electrons’ being trapped, the TD sites should become negatively charged. Due to repulsive interaction between negatively charged dislocation sites and free electrons, extra ionic scattering is expected to occur in the electron transport process, thereby resulting in the decrease of *μ_e_* as observed in the BLSO/STO films (Figure 3b).

It should be noted that, as presented in Figure 3a, BLSO/BSO films have nearly similar carrier concentrations with a nominal dopant concentration (*n*_dop_) (green dashed line). *n*_dop_ is the expected carrier concentration when the donors were fully activated by substitution of La^3+^ for Ba^2+^ ions. This observation supports the good overall stoichiometry of our films, producing nearly expected carrier concentrations as the target materials. Though, a slightly lower *n_e_* could indicate that another source of acceptor-centers other than TDs might exist in the BLSO/BSO films. Paik et al. suggested that the Ruddlesden–Popper crystallographic shear faults might also act as acceptor-centers [16].

**Figure 4:**
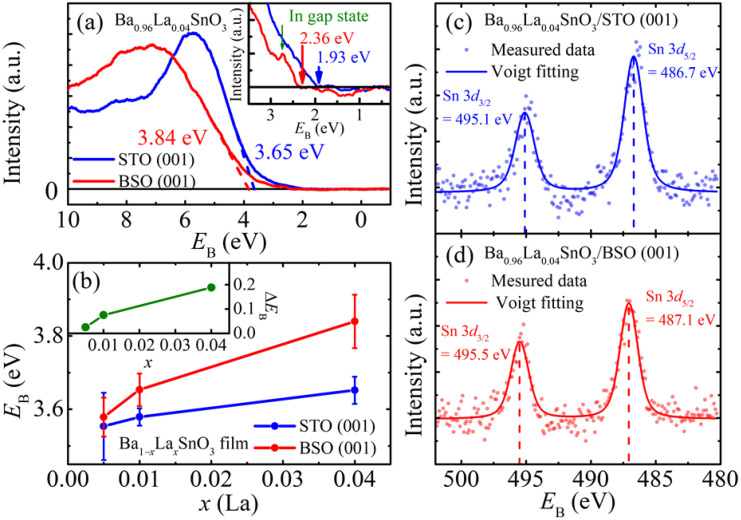
(**a**) The UPS spectra of Ba_0.96_La_0.04_SnO_3_ films grown on STO (001) (blue lines) and BSO (001) (red lines) substrates. The inset shows the same UPS spectrum enlarged near the tail. (**b**) A summary of *E*_B_’s of the valence band maximum (VBM) estimated from the UPS spectra of BLSO films grown on both STO (blue) and BSO (red) substrates. The inset shows the VBM difference between the BLSO films grown on BSO (001) and STO (001) substrates (**c**,**d**) XPS spectra near the Sn 3*d* core level in Ba_0.96_La_0.04_SnO_3_ films grown on STO (001) (blue) and BSO (001) (red). Scattered symbols represent the measured data, and the solid lines are the fitted curves by the Voigt profile. Dashed lines in (**c**,**d**) indicate the peak positions of each spectrum.

To investigate the electronic structure of the BLSO films, both UPS and XPS were employed. The UPS and XPS spectra represent the filled electronic density of states (DOS) as a function of energy below the Fermi energy level (*E*_F_). Due to a low photon energy, ~90 eV, the UPS is an ideal probe to investigate the DOS of the valence band near the *E*_F_. Figure 4a presents the UPS spectra as a function of *E*_B_, showing the valence bands of Ba_0.96_La_0.04_SnO_3_/BSO and Ba_0.96_La_0.04_SnO_3_/STO films comparatively. It is worth noticing in Figure 4a that the valence band of Ba_0.96_La_0.04_SnO_3_/STO is shifted to the lower *E*_B_ as compared to that of Ba_0.96_La_0.04_SnO_3_/BSO. To quantify the valence band shift, the valence band maximum (VBM) was determined as the intercept of the *E*_B_ axis with a linearly extrapolated line on the steeply increasing valence band tails (dashed lines in Figure 4a). The VBM determined by this method represents the *E*_B_ at the valence band edge of the BLSO films. Therefore, the *E*_B_ of VBM should decrease/increase when the valence band is shifted toward/away from the *E*_F_.

As a degenerate semiconductor, the *E*_F_ of the BLSO should be located well inside the conduction band, which is known to be mainly composed of Sn 5*s* orbitals. However, in our UPS study, the filled states from the conduction band structure of the BLSO films could not be identified (see the expanded spectra in the inset of Figure 4a). On the other hand, photoemission spectra corresponding to the filled states from the conduction band of the BLSO films were observed experimentally at *E*_B_~0.5 eV in several hard X-ray photoemission spectroscopy (HAXPES) studies [6,7]. In general, the ratio of photoionization cross-section between Sn 5*s* orbital and O 2*p* orbital decreases exponentially with decreasing photon energy. Considering that the photon energy (~90 eV) used in the UPS measurement is ~10 times lower than that of typical HAXPES measurements, conduction electron states are not likely observable due to the low photoemission intensity of the Sn 5*s* orbital as compared to the O 2*p* orbital.

In the inset of Figure 4a, the UPS spectrum of Ba_0.96_La_0.04_SnO_3_/BSO film shows a peak near the tail region around ~2.75 eV. This peak is suspected to represent the DOS of the in-gap state attributable to various defects in the BLSO film (i.e., La^3+^ donor, oxygen vacancies, etc.) [5]. Even though BLSO/BSO films have no TD, other defects such as oxygen vacancy can be a source of such in-gap states. In contrast, there is no clear peak feature attributable to the in-gap state in the UPS spectrum of the Ba_0.96_La_0.04_SnO_3_/STO film. Since the Ba_0.96_La_0.04_SnO_3_/STO film has TDs as additional defects, its valence band is shifted toward the *E*_F_ so that the edge region of the valence band near 2.75 eV has increased its intensity. Considering the fact that the UPS spectrum of the in-gap state is ~20 times smaller than that of the main valence band region, the in-gap state is expected to be hidden when the in-gap state is located near the valence band edge. Therefore, it is likely that the in-gap state of the Ba_0.96_La_0.04_SnO_3_/STO film is embedded in the band edge spectra.

We have investigated the UPS spectra of other La dopings as well to compare the shifts of VBM in the two sets of BLSO films (Figure S3) and summarized the results in Figure 4b. The VBM shifts of the BLSO films grown on BSO (001) and STO (001) show three major features. First, the *E*_B_ of the VBM in BLSO films generally increases with the increase of *x*. Secondly, BLSO/BSO films exhibit higher *E*_B_’s at the VBMs than BLSO/STO films for all the *x*. Thirdly, the difference of the *E*_B_’s at the VBM between the BLSO/BSO and BLSO/STO films increases with *x* (see the inset of Figure 4b). The first and second behaviors can be simply explained by the *E*_F_ shift. Since the *E*_F_ of the BLSO films increases with *n_e_*, the *E*_B_ of the VBM of BLSO films supposedly increases with *n_e_*. The higher *E*_B_’s of the VBMs observed in the BLSO/BSO films compared to the BLSO/STO films are qualitatively consistent with this explanation. Indeed, the Ba_0.96_La_0.04_SnO_3_/BSO film, which has the highest *n_e_* without TD, exhibits the highest *E*_B_ of the VBM ~3.84 eV among the BLSO films investigated in this work. Moreover, the increasing tendency with *x* observed in the VBM difference between the two film sets (inset of Figure 4b) can also be understood as being due to the *E*_F_ shift. Since the *n_e_* difference between the BLSO/BSO films and the BLSO/STO film increases with *x*, as shown in Figure 3a, the VBM difference between the two sets of BLSO films should also increase.

Although the *E*_F_ shift can explain the valence band shift of the BLSO films qualitatively, it is questionable whether the *E*_F_ shift is sufficient for understanding the effect of TD on the band structure of the BLSO films. To clarify this, we have investigated the core level spectra of the Ba_0.96_La_0.04_SnO_3_ films by XPS. Figure 4c,d shows the Sn 3*d* core level spectra of Ba_0.96_La_0.04_SnO_3_/BSO and Ba_0.96_La_0.04_SnO_3_/STO, respectively. To determine the center positions of the Sn 3*d*_3/2_ and the Sn 3*d*_5/2_ peaks, the spectra were fitted by the Voigt function, which contains both Gaussian and Lorentzian terms to represent instrumental broadening and inherent XPS spectrum, respectively. To determine the instrumental broadening, we measured the XPS spectra of Au metal and fitted the Au 4f peak using the Voigt function with a fixed Lorentzian width (FWHM = 0.29 eV) [25]; the instrumental broadening factor was determined as ~1.26 eV. With the known instrumental broadening factor, the Sn 3*d*_3/2_ and Sn 3*d*_5/2_ core level spectra were fitted by the Voigt profile; Table 1 summarizes the fit results.

According to Table 1, it is unambiguously found that both the Sn 3*d*_3/2_ and Sn 3*d*_5/2_ core levels of the Ba_0.96_La_0.04_SnO_3_/STO are found at the lower *E*_B_’s by ~0.39 eV and ~0.38 eV, respectively, as compared to those of Ba_0.96_La_0.04_SnO_3_/BSO. Moreover, the FWHMs estimated in the XPS spectra of Ba_0.96_La_0.04_SnO_3_/STO and Ba_0.96_La_0.04_SnO_3_/BSO are 1.77 eV and 1.70 eV, respectively. (Note that the same FWHM is assumed for the Sn 3*d*_3/2_ and Sn 3*d*_5/2_ core level spectra.) The FWHM value of the Sn 3*d* spectra in the BLSO/BSO film is indeed in good agreement with that obtained previously in an indium tin oxide film (~1.70 eV), while the FWHM value in the BLSO/STO film is slightly larger (~1.77 eV) [26].

**Figure 5:**
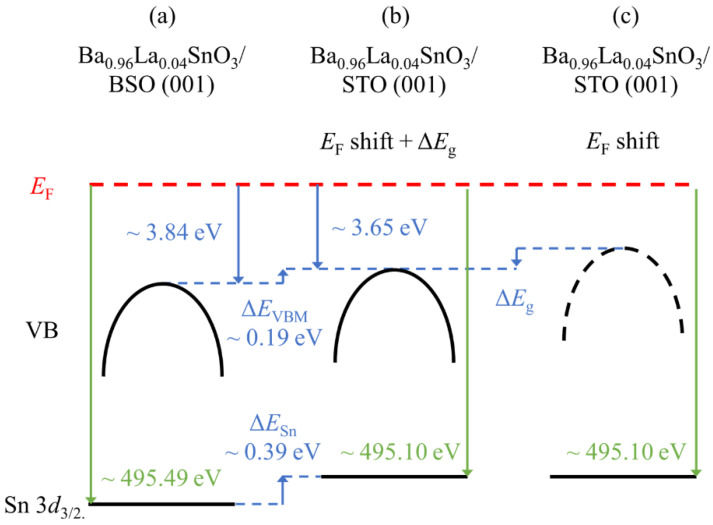
Schematic electronic band structures of (**a**) Ba_0.96_La_0.04_SnO_3_/BSO (001), (**b**) Ba_0.96_La_0.04_SnO_3_/STO (001) with the *E*_F_ shift and the restored band gap (Δ*E*_g_), and (**c**) Ba_0.96_La_0.04_SnO_3_/STO (001) with only the *E*_F_ shift. These band schematics include the valence band (VB), its maximum (VBM), and a Sn 3*d*_3/2_ core level (Sn 3*d*_3/2_).

Since the BLSO films were connected to a common ground in the UPS and XPS measurements, it is natural to align the band structure with respect to *E*_F_. Figure 5 shows the schematic band structures of Ba_0.96_La_0.04_SnO_3_/BSO and Ba_0.96_La_0.04_SnO_3_/STO aligned with respect to *E*_F_. Figure 5a,b shows that both the Sn 3*d*_3/2_ core level and the valence band in the Ba_0.96_La_0.04_SnO_3_/STO film are shifted to lower *E*_B_ by Δ*E*_Sn_ ~0.39 eV and Δ*E*_VBM_ ~0.19 eV, respectively. If the *E*_F_ shift had only occurred with the TDs, Δ*E*_Sn_ and Δ*E*_VBM_ would have been nearly the same as described in Figure 5c. Therefore, experimental observation of different Δ*E*_VBM_ and Δ*E*_Sn_ suggests that the origin of their shift should be explained separately.

One of the most probable explanations for different Δ*E*_VBM_ and Δ*E*_Sn_ is the band gap renormalization effect, which is tied to *n_e_*. The band gap renormalization refers to the shrinking of a band gap proportional to *n_e_*, which is often observed in degenerate semiconductors like the BLSO system [6]. When free electrons are introduced to degenerate semiconductors, conduction bands/valence bands are shifted to a higher/lower *E*_B_, owing to the self-energy effect of added free electrons. Namely, the conduction band is shifted to higher *E*_B_ as the self-energy of added free electrons is negative. For the valence band, however, Coulomb interaction between electrons becomes weakened since the Hartree–Fock exchange is replaced with dynamically screened interaction with added free electrons [27]. As a result, the valence band is shifted to a lower *E*_B_. Therefore, upon *n_e_*’s being increased, the band gap is reduced due to the band gap renormalization, while upon *n_e_*’s being decreased, the bandgap should be restored. In our case, since the TD provides electron trapping sites and clearly reduces *n_e_*, the bandgap should be restored to make the valence band move to higher *E*_B_ in the BLSO/STO films. Hence, due to the combined effect of the lowered *E*_B_ coming from the *E*_F_ shift (0.39 eV) and the restored band gap (0.20 eV), the net VBM shift (0.19 eV) is smaller than in the case with the *E*_F_ shift only (0.39 eV), which results in different energy level shifts in the VBM and the Sn 3*d*_3/2_ core levels, as summarized in Figure 5b.

## 4. Conclusions

The transport, microstructural properties, and electronic band structure of the BLSO films grown on STO (001) and BSO (001) were comparatively investigated to understand the effect of TDs. TDs in the BLSO films play two major roles. First, due to the dangling bonds present in the TDs, they act as electron-traps. Simultaneously, TDs are negatively charged and provide an extra electron scattering center. As a result, TDs make both *μ_e_* and *n_e_* decrease in the BLSO films.

Decreased carrier density attributed to the TDs affects the overall electronic structure of BLSO/STO films. First, *E*_F_ is reduced with reduced *n_e_*. As a result, the *E*_B_’s of the valence band maximum and Sn 3*d* core level become reduced. Secondly, the band gap, which is shrunk in proportion to *n_e_*, is restored with the reduced *n_e_*. This phenomenon is likely to result in the differently valued *E*_B_ lowering in the Sn core level and in the valence band spectra, as observed in the Ba_0.96_La_0.04_SnO_3_/STO (001).

Although only TDs are mainly discussed in this work, other defects, such as oxygen vacancies, can also act as additional electron traps. However, the effects of the other defects on the electronic structure of BLSO films have not been fully understood yet. By combining the result of this study and further research on other defects, we expect that understanding the roles of the various defects on the physical properties of the BLSO films could be helpful to achieve higher quality BLSO films comparable to the BLSO single crystals.

## Figures and Tables

**Table 1:** The Sn 3*d* core level of La 4% doped BLSO film deposited on STO and BSO substrates.

	Sn 3*d*_3/2_	Sn 3*d*_5/2_	FWHM
Ba_0.96_La_0.04_SnO_3_/STO	495.10 ± 0.11 eV	486.69 ± 0.07 eV	1.77 ± 0.06 eV
Ba_0.96_La_0.04_SnO_3_/BSO	495.49 ± 0.11 eV	487.07 ± 0.07 eV	1.70 ± 0.05 eV

## Data Availability

Not applicable.

## Supplementary Materials

The following are available online at https://www.mdpi.com/article/10.3390/ma15072417/s1, Figure S1: Wide range transition electron microscope (TEM) image. Red arrows represent the threading dislocations counted in the density estimation, Figure S2: (a) Schematic diagram of the threading dislocation and (b) sectional diagram of the threading dislocation. Orange line in (b) represent dangling bonds, Figure S3: Ultraviolet photoemission spectra of Ba1–xLaxSnO3 (x = 0.005, 0.01, 0.04) films grown on BSO (001) and STO (001) substrate. Dashed lines indicate linear extrapolation line on the steeply increasing valence band tails.
