# Supplementary material for: Effect of Threading Dislocations on the Electronic Structure of La-Doped BaSnO3 Thin Films
Source: Materials (Basel). 2022 Mar 25;15(7):2417. doi: 10.3390/ma15072417 (PMC9000141; doi:10.3390/ma15072417)
Supplement: Supplementary file 1 [file materials-15-02417-s001.zip › materials-1623706-supplementary.pdf]

Supplementary Material

Effect of Threading Dislocations on the Electronic Structure of La-doped $BaSnO_3$ Thin Films

1. The cross-sectional TEM image in a wide range.

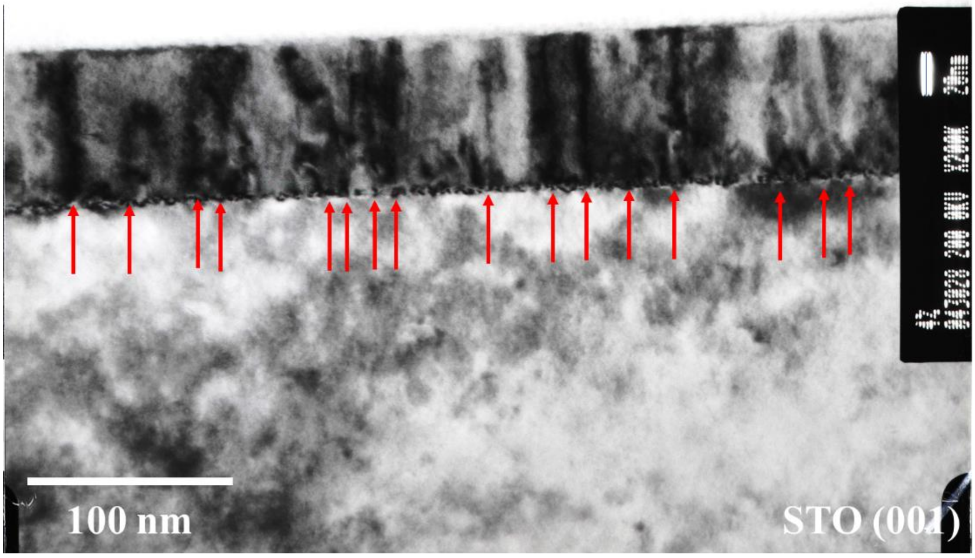

Figure S1. Wide range transition electron microscope (TEM) image. Red arrows represent the threading dislocations counted in the density estimation.

2. Estimation of charge trapped in the threading dislocation

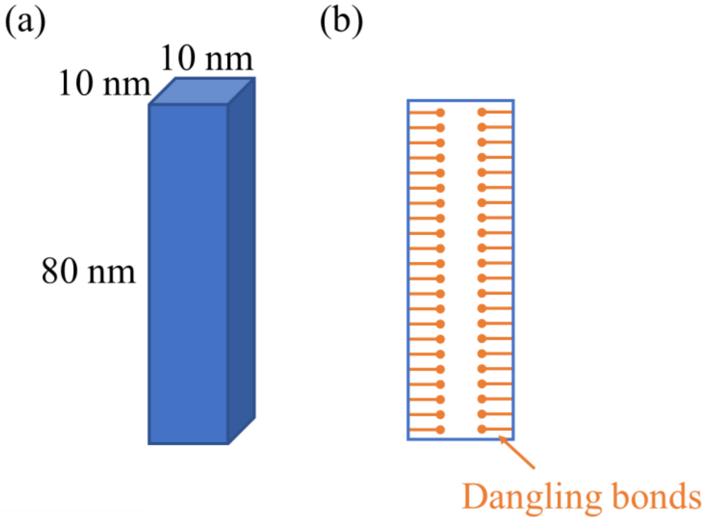

Figure S2. (a) Schematic diagram of the threading dislocation and (b) sectional diagram of the threading dislocation. Orange line in (b) represent dangling bonds.

We have roughly estimated the maximum dangling bond in each threading dislocation by counting unit cells that face the threading dislocations. As t_{film} ~ 80 nm and a typical lateral length of the single threading dislocation, estimated from the TEM image, is ~10

nm wide square, the surface area with the dangling bonds is estimated as ~ 4×10×80 nm^2 = 3200 nm^2. (Figure S2 a) Considering that the lattice parameters of BLSO films are ~ 0.4116 nm, 1.89×10^4 unit cells are estimated to face each threading dislocation. Therefore, assuming each unit cell traps one electron, a maximum of ~1.89×10^4 dangling bonds are expected to be trapped in each threading dislocation.

To compare the estimated maximum electron capacity and trapped electron in each threading dislocation, we have estimated averaged trapped electron per threading dislocation as written below.

$$\frac{Number\ of\ trapped\ carrier}{Number\ of\ theading\ dislocation} = \frac{(n_{dop} - n_e)V_{film}}{N_D A_{film}} = \frac{(n_{dop} - n_e)}{N_D} t_{film}$$

Here, n_{dop} is nominal dopant concentration, which is expected to be fully activated in the BLSO films. And n_e is the measured carrier concentration, N_D is threading dislocation density, V_{film} is the volume of the BLSO film, A_{film} is the area of the BLSO film, and t_{film} is the thickness of the film. Here n_e and N_D is obtained from electrical measurement and TEM image, respectively. In the case of $Ba_{0.96}La_{0.04}SnO_3/SrTiO_3$ (STO) film, it turns out that ~1.38×10^4 electrons are trapped to each single threading dislocation.

According to the estimations above, both values estimated by electrical measurements and counting the number of trapped sites (holes) seem to be reasonably close each other

3. Ultraviolet photoemission sepctra of $Ba_{1-x}La_xSnO_3$ (x=0.005, 0.01, 0.04) films

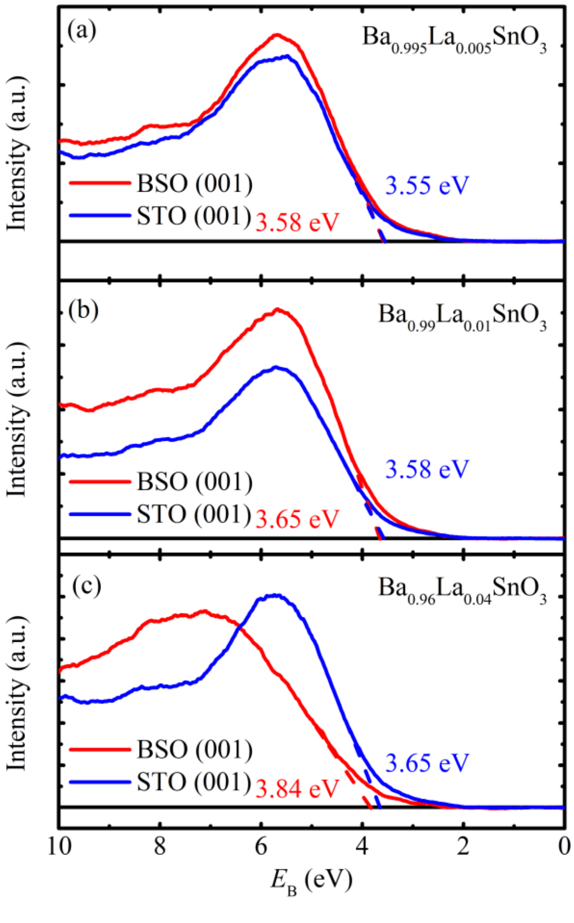

Figure S3. Ultraviolet photoemission spectra of $Ba_{1-x}La_xSnO_3$ (x=0.005, 0.01, 0.04) films grown on BSO (001) and STO (001) substrate. Dashed lines indicate linear extrapolation line on the steeply increasing valence band tails.
